# Effects of Binaural Beat Stimulation in Adults with Stuttering

**DOI:** 10.3390/brainsci13020309

**Published:** 2023-02-11

**Authors:** Dmytro Chernetchenko, Pramax Prasolov, Sam Aganov, Andrii Voropai, Yuliia Polishchuk, Dmytro Lituiev, Eugene Nayshtetik

**Affiliations:** 1 SynthezAI, Research and Development Lab, San Francisco, CA 94105, USA; 2 Department of Physics, Electronics and Computer Systems, Dnipro National University, 49000 Dnipro, Ukraine; 3 UC Berkeley, San Francisco, CA 94720, USA; 4 California Institute for Human Science, Encinitas, CA 92024, USA; 5 University of the Cumberland, Williamsburg, KY 40769, USA; 6 Department of Pedagogy and Psychology, Dnipro National University, 49000 Dnipro, Ukraine

**Keywords:** stuttering, binaural beats, auditory stimulation

## Abstract

In recent decades, several studies have demonstrated a link between stuttering and abnormal electroencephalographic (EEG) β-power in cortex. Effects of exposure to binaural stimuli were studied in adults with stuttering (AWS, *n* = 6) and fluent participants (*n* = 6) using EEG, ECG, and speech analysis. During standard reading tasks without stimulation, in controls but not in the AWS group, EEG β-power was significantly higher in the left hemisphere than in the right hemisphere. After stimulation, the power of the β-band in AWS participants in the left hemisphere increased 1.54-fold. The average β-band power within the left frontotemporal area and temporoparietal junction of the cortex after stimulation in AWS participants shows an increase by 1.65-fold and 1.72-fold, respectively. The rate of disfluency dropped significantly immediately after stimulation (median 74.70% of the baseline). Similarly, the speech rate significantly increased immediately after stimulation (median 133.15%). We show for the first time that auditory binaural beat stimulation can improve speech fluency in AWS, and its effect is proportional to boost in EEG β-band power in left frontotemporal and temporoparietal junction of cortex. Changes in β-power were detected immediately after exposure and persisted for 10 min. Additionally, these effects were accompanied by a reduction in stress levels.

## 1. Background

Stuttering is a speech fluency disorder [ICD-11, World Health Organization, 2019] that is usually diagnosed in infancy, childhood, or adolescence. Stuttering involves impairment in speech fluency and is characterized by frequent repetitions on prolongations of sounds or syllables. Even though 80% of children recover, both with natural development and therapy, the remaining 20% continue to stutter in adulthood, which accounts for 1% of the adult population [1,2,3]. The causes of stuttering are still poorly understood but are usually attributed to speech motor control disruption due to abnormal electrical activity of the brain [4,5], anatomical abnormalities [6,7], genetic and molecular factors [8] stress or psychological trauma [9], or developmental delay [10]. Neurophysiological studies have offered the promise of actionable discoveries regarding stuttering causes [11]. Neuronal electrical activity of the brain is conventionally separated into several frequency bands: δ (0.5–4 Hz), θ (4–7 Hz), α (7–13 Hz), and β (13–30 Hz). Several studies have shown that activity in the electroencephalographic (EEG) β spectrum range, which is commonly heightened in the awake state and focused activities [4], is reduced in individuals with stuttering [5]. EEG and magnetoencephalographic (MEG) studies have shown that in adults who stutter (AWS), compared to the control group, α power is higher [5,12] while β-power is lower during speech production. This may be due to the role of the β-band in coordinating speech planning and execution within the speech-production brain network, as it is known that β-oscillations become more coherent between the bilateral primary motor, premotor, and auditory cortices before speech production [13]. Additionally, EEG and MEG studies suggest that coordination between auditory clues and motor excitation, which is carried out through β oscillations [14], is affected in AWS [15,16,17].

Mersov et al. [18] studied neural oscillations in the speech motor network during preparation and execution of planned speech production in AWS using magnetoencephalography (MEG). Compared to controls, AWS showed stronger *β* suppression in the speech preparation stage, followed by stronger *β* synchronization in the bilateral mouth motor cortex. AWS also recruited the right mouth motor cortex significantly earlier in the speech preparation stage compared to controls [18]. Exaggerated motor preparation is believed to be linked to reduced coordination in the speech motor network in AWS. Korzeczek et al. [19] researched EEG during the spontaneous pre-speech preparation process in AWS. However, they found that stuttering severity is linked to a stronger EEG *β*-power during speech intention. The more severely a person stuttered, the stronger the alpha power in bilateral frontal, and low *β*-power in the mid anterior and right central EEG sensors. AWS with more severe stuttering seem to show stronger maintenance of the current cognitive or sensorimotor state, as stuttering severity was associated with increased beta power. Thus, the findings differ from those described in [18]. This might be explained by differences between [18] and [19] in study designs and differences between spontaneous vs. intentional speech planning studied therein. Alternatively, such differences may suggest that *β*-power suppression occurs differently in AWS with different severity of stuttering, not only during pre-speech formation, but also during the pronunciation process.

A complex pattern of sympathetic–parasympathetic balance emerges in the literature on speech-related stress [20]. On the one hand, speech production [21] and especially speech production in AWS [22] is correlated with higher sympathetic activation. On the other hand, AWS tend to develop a compensatory mechanism by increasing parasympathetic activation [23] and reducing heart rate [24], a phenomenon termed as an autonomous coactivation of ANS [20]. The effect of sympathetic activation reduction by relaxation auditory stimulation of binaural beats with frequency carrier components in *θ*-range of brain activity [25] could accompany the parasympathetic compensatory mechanisms. Thus, we hypothesize that reduction of sympathetic activation and parasympathetic activation may work quite similarly to internal compensation stress reduction mechanisms in AWS and should help to alleviate the severity of stuttering symptoms.

Several methods have been developed before to entrain the brain oscillations. Synchronization of brain activity at the frequency of sensory stimulation and its harmonics in and beyond the corresponding sensory brain areas has been achieved with visual flicker [26], somatosensory tactile stimulation [27], and auditory stimulation. Popular approaches for auditory brain entrainment include isochronous sounds [14,28], monaural beats [29,30,31], and binaural beats [31,32,33,34]. Stuttering reduction using noise and auditory voice feedback treatments has been explored for several decades [35,36]. The feasibility of entraining the *β*-band by isochronous sound exposure has been demonstrated in healthy adults [14] and in AWS [37].

To define the regions of interest within the cortex, we noted that earlier studies found that brain activity EEG patterns normally seen during speech production in non-stutterers were either absent, bilateral in nature, or lateralized to the left hemisphere [38], while in AWS, compensation effects were found in the right hemisphere [39]. Increased gray matter volume in right hemisphere motor [6] and auditory [7] homologues coupled with increased white matter density in the fiber tracts connecting them [7,28,40] have been interpreted as evidence of right hemisphere compensation [41,42]. *β*-band activation in the premotor cortex plays an important role in fluent speech production [39].

Given earlier results suggesting a possible connection between stuttering and the deficit in spectral power of the *β* EEG band [14,43,44] and the lack of activation [28] in speech production centers of the brain, we hypothesized that an increase in *β*-power with binaural beat stimulation [33] might temporarily alleviate disfluency episodes and improve speech quality in AWS.

To test our hypothesis, we exposed adults with stuttering (AWS) and fluent individuals (controls) to proprietary binaural beat soundtracks. Simultaneously, we recorded the EEG, ECG, and speech production. To reveal the influence of auditory stimulation on electrophysiological brain activity, we analyzed the whole EEG spectrum and performed separate analysis for the electrodes group located over the left frontotemporal (LFT) region and temporoparietal junction (LTP) of the cortex. As speech production may be linked to changes in activation of sympathetic ANS, we monitor sympathetic activation in response to auditory stimulation using heart rate variability (HRV).

## 2. Material and Methods

### 2.1. Experimental Design

Here, we performed an uncontrolled interventional study, where we compared characteristics both (a) cross-sectionally between AWS and participants with no speech impairment and (b) longitudinally within each group before and after exposure to binaural beat stimulation. The observation period and data collection were limited to in-lab participation period, except for a brief written follow-up three days after the intervention to inquire about general health adverse events.

### 2.2. Population

Adult volunteers with stuttering (AWS, 6 males; aged 18–38 years; mean age: 28.3 ± 7.1 years) and participants with no speech impairment (controls, 6 males; 26–52 years old; mean age: 32.0 ± 10.1 years) signed informed consent and participated in the study at the laboratory of Faculty of Physics, Electronics and Computer Systems of Dnipro National University. The study was approved by the Ethical Commission of Dnipro National University, protocol #15 from 17 May 2021. All the participants were right-handed and had no history or complaints of hearing difficulties. The stuttering participants received no medications or stuttering therapy during the experiments. None of the control participants reported a history of speech, language, or hearing difficulties.

### 2.3. Exposure: Auditory Stimulation

**Figure 1:**
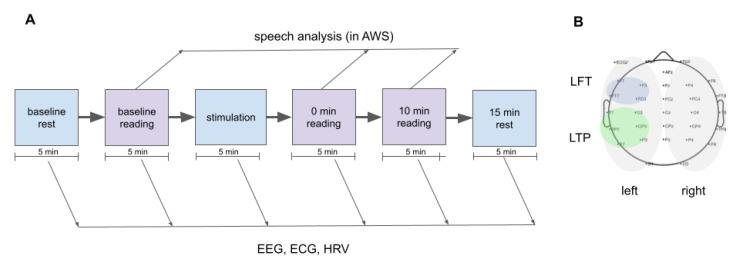
The design of the study. (**A**) Scheme representing the design of the experiments. (**B**) Location of EEG electrodes (32 in total) used during all experiments with highlighted groups of electrodes over the left temporoparietal junction (green on upper topography) and left frontolateral area (blue on upper topography) and groups of electrodes used for the whole-hemisphere analysis (bottom topography; blue for the left and green for the right hemisphere). Abbreviations: EEG—electroencephalographic.

The experimental protocol consisted of 6 stages, 5 min each (Figure 1A): (1) baseline exposure under relaxation and with eyes closed, (2) reading activity pre-stimulation, (3) auditory stimulation, (4) reading task activity immediately after the stimulation, (5) reading task activity after 10 min of relaxation, and (6) resting state measurement with eyes closed. An auditory euphonic binaural beat (BB) stimulus with three spectral components (δ, α, and β) was prepared using a custom generator and post-processing algorithms (United Kingdom Patent Application No. 2116927.1). Ambient royalty-free music with vocals was used as a carrier to specifically target the brain cortical centers responsible for voice recognition (audio recording can be found in the Supplementary Materials in the folder “Stimulus”). To produce a euphonic binaural stimulus [45], we applied a low-pass filter with a cut-off of 170 Hz, which was derived empirically for the chosen music background (to avoid reverberations of sound). The resulting track was normalized in the final stage, and raw stereo audio data were generated. The stimulus used in this study contained binaural components at 3 Hz, 7 Hz, and 21 Hz produced by frequency shift between left and right channel with a low-pass cut-off of the binaural effect at 170 Hz (see Supplementary Figure S1A,B). The spectral characteristics of the resulting auditory output stimuli are shown in Supplementary Figure S1C. The output sound pressure level (SPL) of the stimulus was normalized to the 73 dB level to match the optimal level of auditory binaural stimulation [33] and volume was kept at constant level throughout the stimulation stage. The auditory stimulus was delivered using high-quality wireless Sennheiser momentum true wireless 2 headphones with an output frequency response band in the range of 5 to 33,000 Hz. All participants confirmed that the used SPL would not distract them and described the sound level to be comfortable. The headphones were worn by participants during the whole experiment, but the stimulus was played only during the stimulation phase. During each stage, the EEG and ECG signals were continuously recorded. The reading aloud was assayed in the participants’ native language (Russian) for five minutes. Voice was recorded using a built-in microphone in a MacBook Pro 13” (2015) laptop.

### 2.4. EEG Registration and Signal Processing

A medical-grade digital brain electric activity monitor (CONTEC KT88-3200, S/N 591012399249) was used to collect raw EEG data using conventional wet cap electrodes. Raw data were collected using the proprietary companion software tool CONTEC EEG32. The raw output data were saved in the European EDF+ format. We used 10/20 EEG electrode placements according to the international standard system [46], with reference electrodes A1 and A2 (the electrodes configuration is shown in Figure 1B).

The sampling rate for each channel was 200 Hz and the analogue front-end of the EEG device has 0.1 µV resolution and a 0.016–1000 Hz input signal frequency range. The input data were digitally filtered with a *f* = 0.1–30 Hz band pass filter in order to extract useful EEG signals and exclude electromyographic (EMG) artifacts. Brain electrical activity was post-processed using fast Fourier transform (FFT) followed by power spectrum density (PSD). The noise from electromagnetic interference (EMI) was reduced by applying a notch filter with a cut-off frequency of 50 Hz. Episodes of signals with bad contact of electrodes were excluded from the record by the automatic built-in lead-off detection function of the EEG monitor. Additionally, EOG artifacts were extracted using independent component analysis (ICA) Infomax decomposition algorithm. The absolute power was numerically integrated over each frequency of the following bands: *δ* (0.5–4 Hz), *θ* (4–7 Hz), *α* (7–13 Hz), and *β* (13–30 Hz) over each electrode’s position (32-electrode EEG measurement). Consequently, an average of 32 channels was used for the statistical analysis of the spectral power. The spatial distribution of the PSD calculated using a standard formula was visualized as a heatmap for each EEG spectral band. To obtain the power of electrical brain activity in areas associated with speech production processes, we calculated the mean average power at the left temporoparietal junction (anatomically associated with Wernicke’s area) within electrodes C3, CP3, P3, P7, TP7, and T7, and the left frontotemporal area within electrodes F3, FC3, F7, and FT7 (anatomically associated with Broca’s area). Topographies, compressed spectrum graphs, and trend graphs were produced using open-source EEGLAB v.2021.1 software.

### 2.5. ECG Registration and Signal Processing

Electrocardiographic (ECG) recordings were conducted using a CONTEC 8000GW device (HW S/N: 39124881923) with four wet electrodes placed on the limbs to obtain a conventional six-lead ECG system (*I*, *II*, *III*, *avL*, *avR*, and *avF*). The input analog ECG signal was amplified with 1000x gain by a low-noise analog front-end. To remove artifacts, ECG-signal segments with poor contact of electrodes were detected and removed using built-in AC-current lead-off detection. The input signal was low-pass filtered with a Butterworth IIR filter of order *n* = 8 and cut-off frequency *f* = 30 Hz to prevent aliasing and exclude electromyographic (EMG) artifacts. Baseline drift was removed with a high-pass filter Butterworth IIR filter of order *n* = 8 and cut-off frequency *f* = 0.67 Hz. The sampling frequency was 1000 Hz, while pass-band attenuation was taken at 1 dB. The influence of EMI was reduced by applying a notch filter with a cut-off frequency of 50 Hz. Raw ECG data were exported to HL7 aECG standard XML format. In order to detect QRS complexes and extract R-peaks in ECG signals, the Pan–Tompkins algorithm [47] was used. The algorithm includes the signal preprocessing stages and R-peaks detection, which were implemented in Python programming language.

The state of the autonomic nervous system (ANS) and stress level were assessed by heart rate variability (HRV) using a set of standard metrics calculated from ECG RR intervals (heart beat-to-beat intervals) brought forth by the European Society of Cardiology and North American Society HRV Standards (“Heart Rate Variability,” 1996): meanRR, mean heart rate, standard deviation of NN intervals (SDNN), root mean square of successive differences (RMSSD), low-to-high frequency ratio (LF/HF), low frequencies power (LF), high frequencies power (HF), and Baevsky stress index. The Baevsky stress index (SIdx) is a measure of HRV reflecting cardiovascular system stress. High values of SI indicate reduced variability and high sympathetic cardiac activation. SIdx was computed according to the formula (Baevsky, R.M.; Berseneva, A.P., n.d.):

(1) $$SIdx=\frac{AMo\times 100\%}{2\;Mo\times MxDMn},$$

where *AMo* is the mode amplitude presented in percentage points, *Mo* is the mode (the most frequent RR interval), and *MxDMn* is the variation scope reflecting the degree of RR interval variability. Mode *Mo* was simply calculated as the median of the RR intervals. The *AMo* was obtained as the height of the normalized RR interval histogram (bin width 50 ms) and *MxDMn* as the difference between the longest and shortest RR interval values. To make SIdx less sensitive to slow changes in mean heart rate (which would increase the *MxDMn* and lower *AMo*), the very low frequency trend is removed from the RR interval time series using the smoothness priors method [48].

### 2.6. Speech Quality Assessment

Speech recordings were evaluated by speech therapists for a range of disfluencies, such as repetitions of syllables, whole words, sound prolongations, and blocks. Each audio recording was scored blindly by a speech therapist (a participant ID was provided but neither experimental stage nor participants’ cohort). Disfluencies were measured in terms of the number of repetitions (separately for sounds, syllables, and words), prolongations, and blocks. The total number of disfluencies per experiment was normalized to the total number of words read and presented as disfluency rate. As the texts differed from one stage of the experiment to the other (each time a new unfamiliar text was proposed for reading to exclude memorization effect), it was necessary to calculate the relative number of disfluencies for each measurement. The same set of texts was provided to all participants at respective stages of the experiment. Speech rate was estimated as words read per minute. At the end of the experiment, participants rated the pleasantness of auditory stimulation and its effect on speech abilities as well as their overall stress before and after stimulation with available options: easier to speak after stimulation; no change; and harder to speak after stimulation.

### 2.7. Statistical Analysis

To examine the effect of treatment for each cohort (AWS, controls) on physiological readouts while accounting for repeated experiments within participants, we applied linear mixed effects [49] models (LMM). Every set with physiological raw data was processed and scored blindly by automated script written in Python programming language.

All statistical analyses were performed using the R programming language. We researched the relationships between changes in spectrum power of brain cortex electrical activity after stimulation and disfluency rate of speech. To assess relationships across these variables, non-parametric tests (i.e., Spearman’s rank) were used where variables were not normally distributed. The effects due to participants’ individual physiology were treated as a varying intercept random effect, effects due to the experiment were treated as random effects nested within the participants’ effects, and effects due to the cohort or treatment were treated as fixed effects. Analysis was performed using lme4 and lmerTest libraries in R software, and the significance of the fixed effects was reported. Speech characteristics were compared using the Wilcoxon t-test for a single (first) experiment per participant.

## 3. Results

### 3.1. Auditory Stimulation Results in Reduction of Stress Level as Monitored by ECG

**Figure 2:**
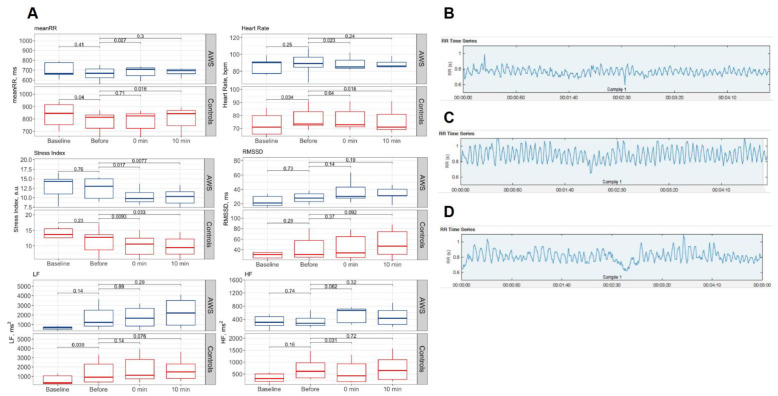
ECG-based markers of stress during reading tests. (**A**) HRV markers (meanRR, heart rate, stress index, RMSSD, LF, and HF) are shown for AWS and control group in baseline rest state, reading before the stimulation, immediately after (0 min), and 10 min after the binaural beats stimulation. RR intervals deviation with time demonstrate HRV characteristics before stimulation (**B**), right after stimulation (**C**), and 10 min after stimulation (**D**). Note an increase in amplitude and higher frequencies alongside with reduction in regularity of oscillations exemplifying physiologic relaxation. Abbreviations: ECG—electrocardiography, HRV—heart rate variability, AWS—adults with stuttering; RMSSD—root mean square of successive differences, LF/HF—low-to-high frequency ratio.

To assess the reaction of AWS and controls on reading tasks, we compared HRV parameters in the resting state and during the initial reading. The reaction of AWS and controls was different. In controls, unlike AWS, heart rate and LF power during the reading increased relative to the baseline resting state (by 1.04 ± 0.06 *p* = 0.03 and 2.51 ± 1.69 *p* = 0.03-fold, respectively, in Wilcoxon test). Next, we evaluated the effect of auditory stimulation on general stress level, heart rate, and HRV parameters to elucidate ANS state. A significant decrease was observed in the heart rate immediately after stimulation compared to the baseline (*p* = 0.024 in Wilcoxon tests). After 10 min, the heart rate stayed significantly lower only in the control group (*p* = 0.01 in Wilcoxon test). Moreover, the Baevsky’s stress index was down in both AWS and control group relative to the baseline both immediately after the exposure (by 1.16 ± 0.12 *p* = 0.01 and 1.26 ± 0.19 *p* = 0.03-fold, respectively in Wilcoxon test) and 10 min later (*p* = 0.01 and *p* = 0.03 in Wilcoxon test, see Figure 2A). No significant difference was found in the HRV RMSSD after stimulation. An example of heart rate modulation during the experiment is shown by an ECG series for one AWS participant before, immediately after, and 10 min after stimulation (see Figure 2B–D, respectively).

Cross-sectional analysis shows that meanRR was 0.85x and 0.82x lower in AWS than in fluent participants in baseline and during the stimulation, respectively (*p* = 0.03, see Supplementary Table S2).

### 3.2. AWS Display Distinct Spatial Signatures of Brain Activation during Speech Production

To evaluate the baseline electrophysiological differences between AWS and controls, we measured EEG characteristics for both groups. No significant difference in *β*-power averaged across all electrodes was found between the control and AWS cohorts in the baseline rest state (*p* = 0.43 in Wilcoxon test, see Supplementary Figure S2A,B). The total power in the *β*-band in the control group was significantly higher in the left hemisphere than in the right hemisphere (*p* = 0.03 in Wilcoxon test) during the baseline state measurements. However, electrical activity in the AWS during the resting state in the left and right hemispheres was the same (*p* = 0.38 in Wilcoxon test).

Next, during the reading task in the baseline state, the average power of the *β*-band did not differ significantly between the AWS and control groups (*p* = 1.0 in Wilcoxon test). Left–right hemispheric asymmetry in *β*-band power was observed in the control group (*p* = 0.03 in Wilcoxon test) but not in the AWS group (*p* = 0.74 in Wilcoxon test). Detailed results of the hemispheres’ electrical activity distribution during the experiment are shown in Supplementary Figure S2A,B and Table S1.

The *β*-power in LTP junction and LFT cortex area during the reading task did not significantly increase neither in AWS (*p* = 0.64 in Wilcoxon test, *p* = 0.54 in Wilcoxon test) nor in the control group (*p* = 0.06 in Wilcoxon test in controls, *p* = 0.15 in Wilcoxon test in controls) relative to the baseline rest state. However, the relative average power of the *β*-band in the LTP junction and LFT area correlated across participants in the control group (Spearman *ρ* = 0.89, *p* = 0.03, Supplementary Figure S2B) and AWS group (Spearman *ρ* = 0.9, *p* = 0.05, see Supplementary Figures S6A and S7A).

### 3.3. Auditory Stimulation Enhances β Spectral Power and Leads to Spatial Redistribution of Brain Activity

To assess the effectiveness of the auditory binaural beat stimulation in enhancing modulated frequencies of brain electrical activity, we recorded and analyzed EEG activity before, during (see Supplementary Figure S4A), and after stimulation, at rest, and during reading activity, as shown in Figure 1, and analyzed the overall power of the *β*-band, as well as each hemisphere, and within cortex projections related to the speech production.

**Figure 3:**
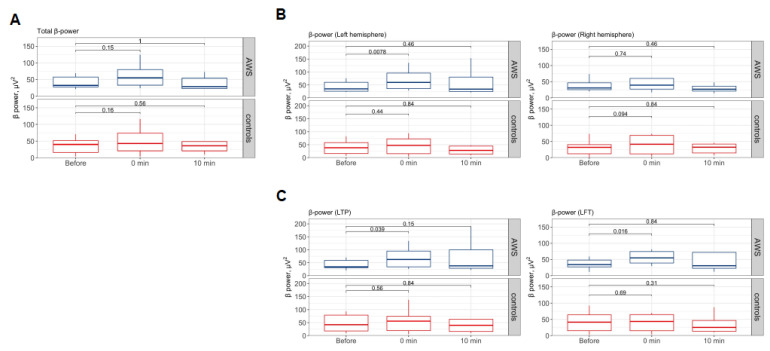
EEG *β*-power change at the different stages of experiment. (**A**) EEG *β*-waves in reading experiments in AWS (**top**) and control group (**bottom**) averaged across all 32 electrodes. (**B**) Power in *β*-band for left and right hemispheres (**left** and **right**, respectively) during the reading activities in AWS and controls. (**C**) Power in *β*-band averaged across electrode groups located over the LTP junction (**left**) and LFT area during the reading activities in AWS and controls. Abbreviations: EEG—electroencephalography, AWS—adults with stuttering; LTP—left temporoparietal junction; LFT—left frontotemporal area.

**Figure 4:**
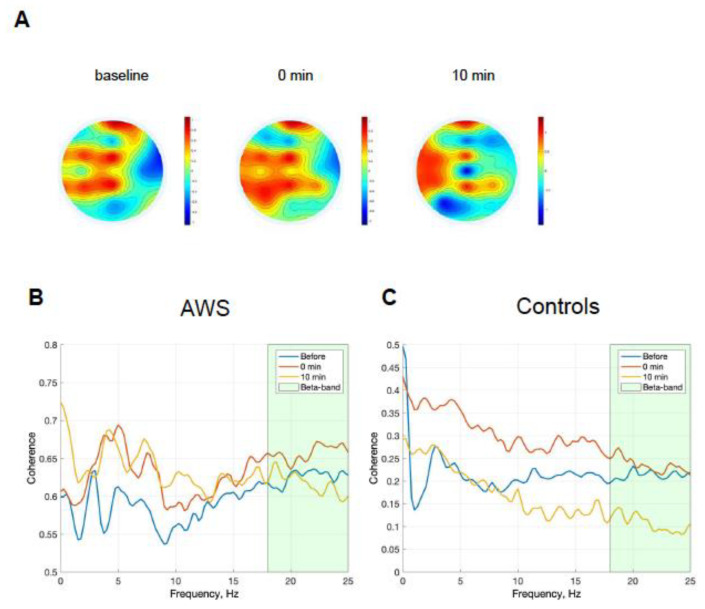
Topography and frequency specificity of binaural beats stimulation-induced changes. (**A**) Topographical distribution of *β*-power before, after, and 10 min after the stimulation. (**B**) Coherence between LTP junction and LFT area during speech in AWS before, immediately after, and 10 min after stimulation. (**C**) Coherence between LTP junction and LFT area during speech in controls before, immediately after, and 10 min after stimulation Abbreviations: AWS—adults with stuttering; LTP—left temporoparietal junction; LFT—left frontotemporal area.

The overall absolute *β*-power in the control group was insignificantly increased by 1.4-fold after stimulation (*p* = 0.16 in Wilcoxon test) and stayed at 1.2-fold 10 min after compared to the baseline reading (*p* = 0.56, Wilcoxon test). The overall *β*-power in the AWS insignificantly increased by 1.32-fold after stimulation (*p* = 0.15 in Wilcoxon test) and remained at 1.14-fold 10 min after stimulation (*p* = 1.0 in Wilcoxon test; see Figure 3A) compared to the baseline reading. Detailed analysis of spatial distribution showed that after stimulation, the power of the *β*-band in AWS participants in the left hemisphere increased 1.63-fold (with *p* = 0.01 in Wilcoxon test), while changes in the right hemisphere activity were not significant (*p* = 0.74 in Wilcoxon test, see Figure 3B). In the control group, no significant changes in *β*-power were observed in either brain hemisphere after stimulation (*p* = 0.44 in Wilcoxon test for left; *p* = 0.09 in Wilcoxon test for right hemisphere, see Figure 3B). Spatial changes were visualized as differences in the topography of grand-averaged normalized *β* brain activity during text reading after stimulation (see Figure 4A).

Next, we analyzed the average *β*-band power within the LFT and LTP junctions. After the stimulation, the average power of the *β*-band within LTP projection in AWS participants increased by 1.74-fold after stimulation (*p* = 0.03 in Wilcoxon test) and stayed at 1.65-fold 10 min after stimulation (*p* = 0.15 in Wilcoxon test compared to the baseline). After stimulation, the average power of the *β*-band within the LFT area in AWS participants increased by 1.72-fold after stimulation (*p* = 0.01 in Wilcoxon test) and stayed at 1.61-fold 10 min after stimulation (*p* = 0.84 in Wilcoxon test compared to the baseline). Coherence analysis between the electrodes electrical activity associated with LFT area and LTP junction in AWS cohort showed that average coherence within *β*-band before stimulation was equal to 0.628 ± 0.009 (I = 64.44, I_beta_ = 7.54), after stimulation 0.66 ± 0.01 (I = 60.73, I_beta_ = 7.91), and 10 min after 0.606 ± 0.01 (I = 62.91, I_beta_ = 7.27) (see Figure 4C). At the same time, in the control group, the coherence within *β*-band before stimulation was 0.21 ± 0.01 (I = 21.29, I_beta_ = 2.61), after stimulation 0.23 ± 0.01 (I = 32.23, I_beta_ = 2.75), and after the next 10 min 0.1 ± 0.01 (I = 22.64, I_beta_ = 1.24) (see Figure 4D). Additionally, we observed a cross-sectional correlation between the activity of the two areas in both AWS (see Supplementary Figure S6A) and control (see Supplementary Figure S6B) groups immediately after the stimulation (Spearman *ρ* = 1.0, *p* = 5 × 10^−5^, in AWS, and *ρ* = 0.94, *p* = 0.01, in controls, respectively) and 10 min later (Spearman *ρ* = 0.86, *p* = 0.01, in AWS, and *ρ* = 0.97, *p* = 0.001, in controls, respectively). Cross-sectional correlations for each patient are shown in Supplementary Figure S7. No cross-sectional difference was observed in β-power between AWS and fluent adults as assessed by total power, hemispheric, LFT, or LTP projection area components (Supplementary Table S3).

### 3.4. Auditory Stimulation Leads to Improvement of Speech Quality and Conditional β-activation in Speech Centers in AWS

**Figure 5:**
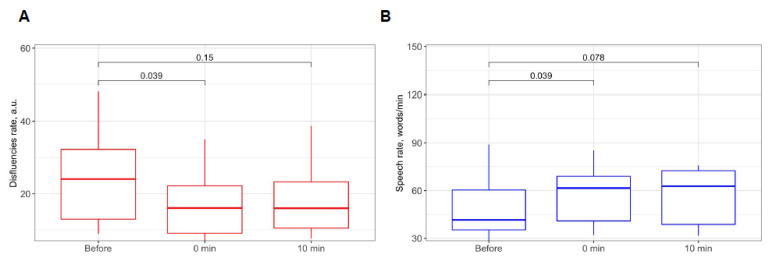
Speech disfluency and speech rate during experiments. (**A**) Speech disfluency rate before, after, and 10 min after binaural beats stimulation. (**B**) Speech rate before, after, and 10 min after binaural beats stimulation.

To investigate the effect of auditory stimulation on speech quality, we recorded speech samples before and after the stimulation (Figure 1). The speech rate (words per minute) and rate of disfluencies (the ratio of disfluencies to the total number of words uttered) were counted in the AWS group by a speech pathologist. The rate of disfluencies dropped after the stimulation (median 74.70% of the baseline rate with 90% CI: 37.11–98.16%, *p* = 0.04 in paired Wilcoxon test, see Figure 5A), but there was no significant difference from the baseline 10 min later (median 65.51% of the baseline rate, 90% CI: 39.15–93.70%, *p* = 0.15; see Figure 5B). Similarly, speech rate increased immediately after stimulation (median 133.15% of the baseline rate, *p* = 0.04), but was not significantly different from the baseline 10 min later (median 126.63% of the baseline rate, *p* = 0.08). Immediately after stimulation, six out of the six participants noted the subjective ease of reading the text aloud (short questionnaire described in the Materials and Methods section). Speech recordings showed a smoother, clearer, and faster text pronunciation for AWS after stimulation (see the link on records in the Supplementary Materials).

### 3.5. Improvement of Speech Quality Is Correlated to the Specific Brain Activity Patterns

**Figure 6:**
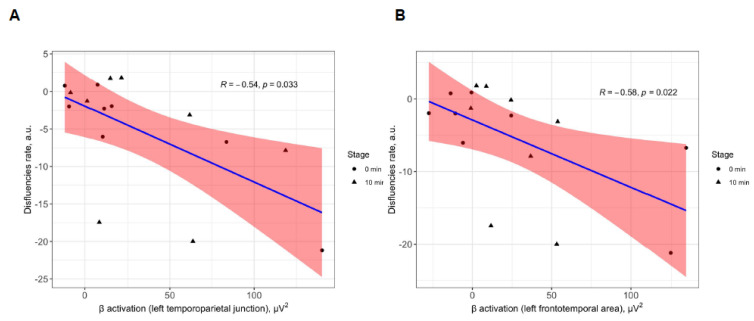
Correlation between speech disfluencies and β-power over the speech-relevant centers after binaural beats stimulation (0 min and 10 min after). (**A**) Correlation between disfluency rate per AWS participants and β-band power average across electrodes over the LTP junction. (**B**) Correlation between disfluency rate per AWS participants and β-band power average across electrodes over the LFT area. Abbreviations: AWS—adults with stuttering; LTP—left temporoparietal junction; LFT—left frontotemporal area.

As speech improvement is expected to be mediated by activation of the LFT area and LTP junction, we analyzed the correlation between changes in the rate of disfluencies and activation in the respective areas. To elucidate the contribution of auditory stimulation-induced changes in electrical activity to changes in speech fluency, we compared changes in *β*-band power in the LFT and LTP junction before and after stimulation to the disfluency rate. Improvement in fluency after stimulation and 10 min after compared to the reference reading stage correlated with an increase in the power of the *β*-band in both LTP junctions (Spearman *ρ* = −0.54, *p* = 0.03, see Figure 6A) and LFT area (Spearman *ρ* = −0.58, *p* = 0.02, see Figure 6B).

When comparing speech fluency between the AWS participants who responded with an immediate increase in *β*-power within the speech centers upon auditory stimulation (*n* = 4, average increase by 2.3-fold) to the rest of the AWS participants (*n* = 2), the former displayed an improvement in speech fluency both immediately (30.03%, *p* = 0.03 in the paired Wilcoxon test) and after 10 min (25.38%, *p* = 0.03 in the paired Wilcoxon test). Additionally, in the responder sub-group, the increase in *β*-power persisted after 10 min (1.77-fold relative to the baseline reading stage, *p* = 0.03 in the paired Student’s t-test, *p* = 0.05 in the paired Wilcoxon test). This suggests that improvement in speech quality may be mediated by activation of the *β*-frequency band in speech centers, and the change in spectral power of the *β*-band may serve as a surrogate variable predictive of functional response in speech quality.

## 4. Discussion

In this study, we assessed the responses of adults with and without stuttering to auditory stimulation with binaural beats using a broad range of physiological metrics.

A hypothesis that drove our efforts stated that participants with stuttering may exhibit lower *β*-power than controls [14,43,44]. We were unable to confirm this hypothesis in our cohort, potentially due to the low number of participants. However, we observed hemispheric asymmetry in *β* activity in controls but not in AWS, which is in agreement with observations in children who stutter [11]. Next, we were able to induce an increase in *β*-power during reading activity in AWS but not in controls by means of auditory binaural beat stimulation. This did not create a significant left–right asymmetry in participants with stuttering. However, the increase in *β*-power in the AWS was significant only in the left hemisphere. This is a desirable effect, as the speech centers localize to the LTP junction and LFT cortex area. Furthermore, we observed that upon auditory stimulation, the *β*-power increased in left projections in AWS but not in controls.

Next, we demonstrated that auditory stimulation improved speech quality. The effectiveness of *β*-range BB stimulation in improving speech quality in AWS agrees with earlier studies, suggesting a link between *β* deficit and stuttering [5,12,43]. Moreover, our results suggest that there is a dose-effect relationship between speech improvement and the increase in EEG *β*-power. As a further step, we need to explore the effect of stimulation on a larger group of AWS with a longer follow-up period.

Stuttering episodes are often accompanied by stress and anxiety [50], which is another common target of stuttering therapy [51]. In this study, we probed stress levels by monitoring participants’ heart rate and heart rate variability (HRV) by ECG RR intervals. We showed that the response to speech-related stress in AWS and controls is different. During the reading task, controls show significant increase in heart rate and sympathetic activation (increasing of LF spectrum power), while AWS do not. This substantiates the existence of compensatory mechanisms in AWS [20]. Additionally, our stimulation reduced HR and increased HRV in AWS. We observed a significant drop in the stress index after exposure to the stimuli in participants with stuttering and controls, suggesting a reduction in stress levels. This is in agreement with earlier work showing that brain waves can be entrained with BB [33,52], which may cascade into other physiological functions, such as HRV [25,53].

Among the limitations of this study, one limitation is that we did not have the resources to include a potentially blinded sham control for binaural beat stimulation. As a further step, we plan to reproduce results with sham audio stimuli without BB and study the effect of both amplitude and exact frequency of binaural shift in *β*-range of brain waves (currently set to 21 Hz) on fluency improvements. Other tone frequencies should be further investigated because the optimal resonant frequency can be individualized for different patients. The hypothesis of calibration of the modulated frequency may depend on individual brain electrical activity and may be reflected in speech phonetic patterns. Cross-over design and placebo trials of the experiment should be conducted to clarify the effects of the stimulus that do not depend on groups of participants. The long-term effects of exposure, including possible habituation or long-term exposure, as well as the impact on participants’ general well-being, should be further investigated. The duration and effect of carrier music parameters, as well as variations in the binaural shift frequency within the conventional range 3 to 30 Hz [31,32,33,34] on the effect duration, may be investigated in more detail in future research.

No adverse effects were observed in this study. The participants did not experience negative or irritating effects or fatigue after 5 min of BB stimulation. Thus, longer stimulation may also be considered because of the non-invasiveness and absence of side effects of the proposed approach. In addition, such an auditory entertainment approach can be used in a multi-day course with several sessions.

An in-depth understanding of the effects of the auditory BB stimulation and its capability to temporarily reduce stuttering symptoms may be useful for end-user applications. The findings of this work may be used for the design of digital therapeutic techniques for the treatment of speech disfluencies.

## Data Availability

Data may not be shared due to participant confidentiality concerns.

## Supplementary Materials

The following supporting information can be downloaded at: https://www.mdpi.com/article/10.3390/brainsci13020309/s1, Figure S1: Spectral characteristics of auditory stimulus; Figure S2: Changes in β power during the experiment; Figure S3: Changes in relative power averaged across all electrodes for different frequency bands (α, β, δ and θ) for reading tasks before, after and 10 minutes after binaural beats stimulation; Figure S4: Time-resolved topography of β-power during binaural beats stimulation; Figure S5: Snippets of the raw speech signals; Figure S6: Cross-sectional correlation between normalized activity the left temporoparietal (LTP) projection and left frontotemporal (LFT) area; Figure S7: Cross-sectional correlation between activity the left temporoparietal (LTP) junction and left frontotemporal (LFT) area; Table S1: EEG β-power values during the experiment, separately averaged for the electrodes of the left and right hemispheres; Table S2: ECG parameters cross-cohort comparison presented as p-values of unpaired Wilcoxon test; Table S3: Comparison of EEG power between AWS and fluent participants (Wilcoxon test).
