# Supplementary material for: Effects of Binaural Beat Stimulation in Adults with Stuttering
Source: Brain Sci. 2023 Feb 11;13(2):309. doi: 10.3390/brainsci13020309 (PMC9954735; doi:10.3390/brainsci13020309)
Supplement: Supplementary file 1 [file brainsci-13-00309-s001.zip › brainsci-2121959-supplementary.pdf]

SUPPLEMENTAL INFORMATION

Supplementary Materials, including audio and video records demonstrating the effect of auditory stimulation, can be found online at the following link:
https://drive.google.com/drive/folders/1h3Jg7hcAV5WNuRwLAHfTrzqOmTMIQLfS?usp=sharing

Supplementary Methods.

ECG R-peaks detection algorithm.

In order to detect QRS complexes and extract R-peaks in ECG signals, the Pan-Tompkins algorithm (Pan et al., 1985) was used. Algorithm includes the signal preprocessing stages and R-peaks detection and were implemented in Python programming language. As a first step, a band-pass filter is applied to increase the signal-to-noise ratio (SNR). Next, a derivative filter is applied to provide information about the slope of the QRS. The filtered signal is squared to enhance the dominant peaks (QRSs) and reduce the possibility of erroneously recognizing a T wave as an R peak. Then, a moving average filter is applied to provide information about the duration of the QRS complex. In order to detect a QRS complex, the local peaks of the integrated signal are found (R-peaks). A peak is defined as the point (fiducial mark) in which the signal changes direction (from an increasing direction to a decreasing direction). Each fiducial mark is considered as a potential QRS. To reduce the possibility of wrongly selecting a noise peak as a QRS, each peak amplitude is compared to a threshold that takes into account the available information about already detected QRS (signal threshold) and the noise level (noise threshold). The algorithm takes particularly into consideration the possibility of a false detection of T waves. So, if a potential QRS falls up to a 160 ms window after the refractory period from the last correctly detected QRS complex, the algorithm evaluates if it could be a T wave with particular high amplitude. The algorithm takes into account the possibility of setting too high values of adaptive thresholds and miss R-peaks, to avoid this situation the search back approach for missed QRS applied based on the average RR is computed in two ways to consider both regular and irregular heart rhythm.

Supplementary Table S1. EEG β-power values during the experiment, separately averaged for the electrodes of the left and right hemispheres. P-value for the Wilcoxon test of difference between the left and right hemispheres is provided.

**Condition**	**Cohort**	**Left (μV^2)**	**Right (μV^2)**	**p-value**
Baseline reading	AWS	43.5 ± 21.9	54.8 ± 21.9	*0.74*
After stimulation	AWS	71.1 ± 42.1	53.8 ± 42.8	*0.19*
Post effect	AWS	67.4 ± 66.8	48.2 ± 61.0	*0.38*
Baseline reading	controls	39.9 ± 29.3	31.1 ± 26.1	***0.03****
After stimulation	controls	46.5 ± 36.6	53.5 ± 56.9	*1.00*
Post effect	controls	50.5 ± 64.1	28.6 ± 16.7	*0.43*

Supplementary Table S2. ECG parameters cross-cohort comparison presented as p-values of unpaired Wilcoxon test

Measurement	Baseline	Reading	Stimulation	0 min after stimulation	10 min after stimulation
meanRR, ms	***0.03****	0.09	***0.03****	0.21	0.09
SDNN, ms	0.84	1.00	0.31	0.84	1.00
RMSSD, ms	0.31	0.56	0.09	0.84	0.31
HR, bpm	***0.03****	0.09	***0.03****	0.15	0.10
vLF	0.09	1.00	0.15	0.68	0.84
LF	0.68	0.84	0.68	1.00	0.43
HF	0.43	0.31	0.43	0.84	0.56
LF/HF	0.68	0.09	0.21	0.68	0.15
TotalPower	0.68	1.00	0.84	0.68	0.68
StressIndex	1.00	0.90	1.00	0.80	1.00

Supplementary Table S3. Comparison of EEG power between AWS and fluent participants (Wilcoxon test)

Measurement	Baseline	Reading	Stimulation	0 min after stimulation	10 min after stimulati on
Average β *power*	0.43	1.0	0.68	0.21	0.21
LFP projection	0.84	0.84	0.68	0.43	0.21
LTP projection	1.00	1.00	0.56	0.56	0.68
Left	0.68	1.00	0.56	0.56	0.56
Right	0.43	0.84	0.43	0.84	1.00

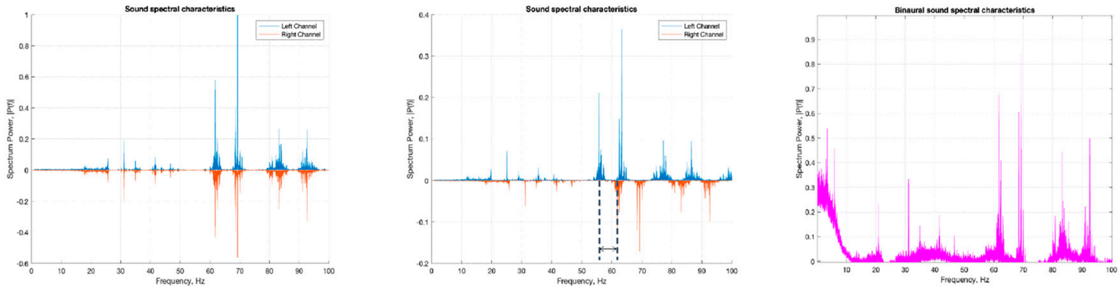

Supplementary Figure S1. Spectral characteristics of auditory stimulus

A. Spectrum of an input signal.

B. Spectrum of resulting signal with single target frequency (7 Hz) shift.

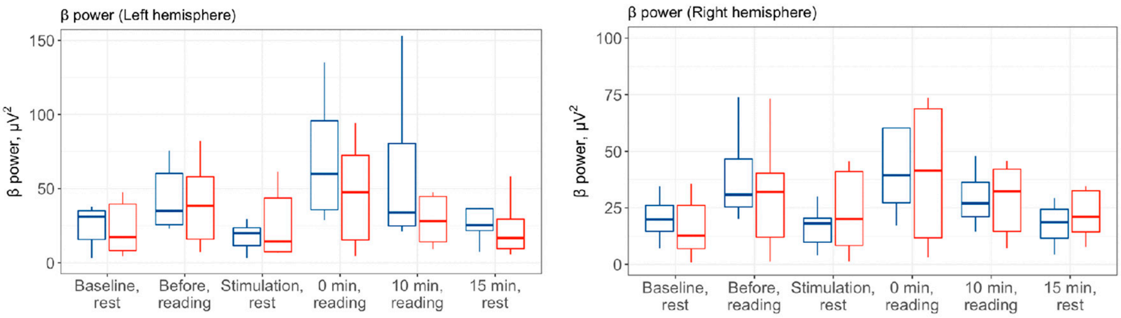

Supplementary Figure S2. Changes in *β* power during the experiment.

A. *β* power spectrum for left hemisphere in both cohorts during all stages of experiment.

B. *β* power spectrum for right hemisphere in both cohorts during all stages of experiment.

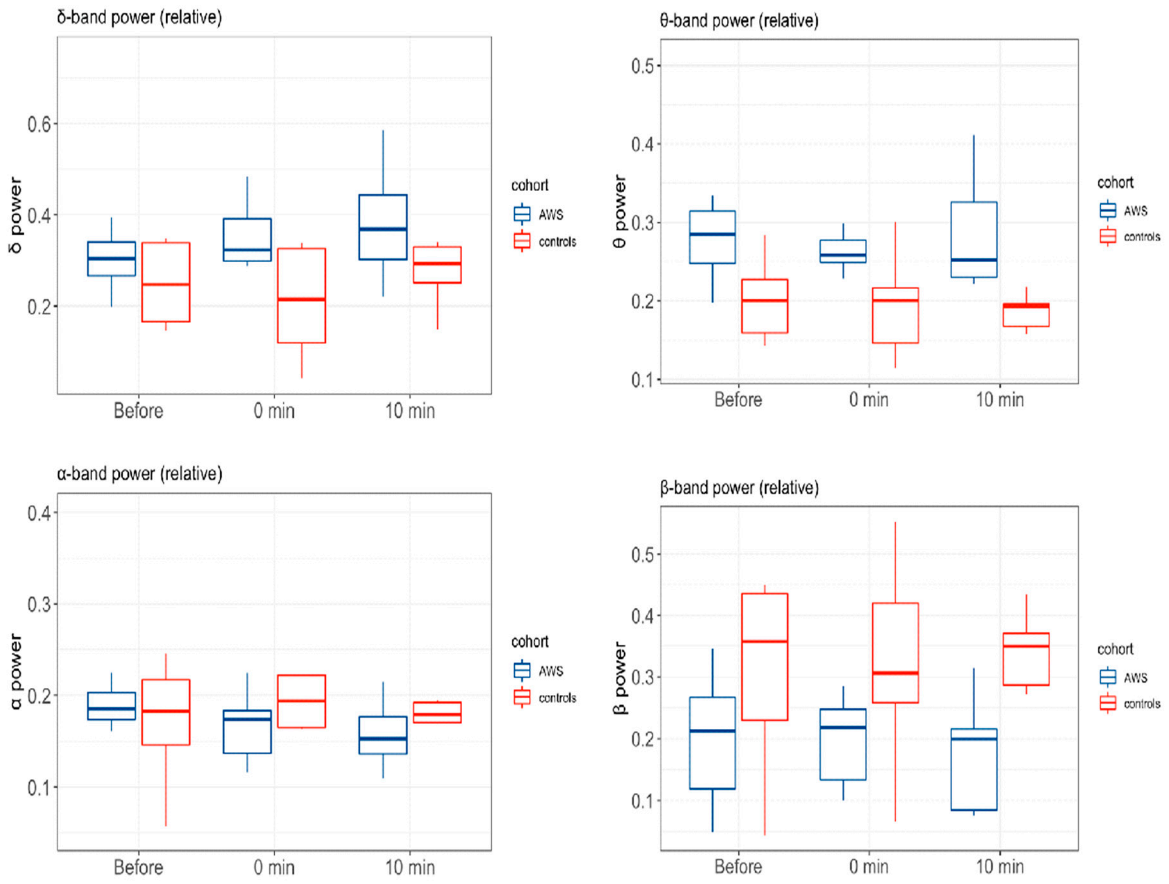

Supplementary Figure S3. Changes in relative power averaged across all electrodes for different frequency bands (*α, β, δ* and *θ*) for reading tasks before, after and 10 minutes after binaural beats stimulation.

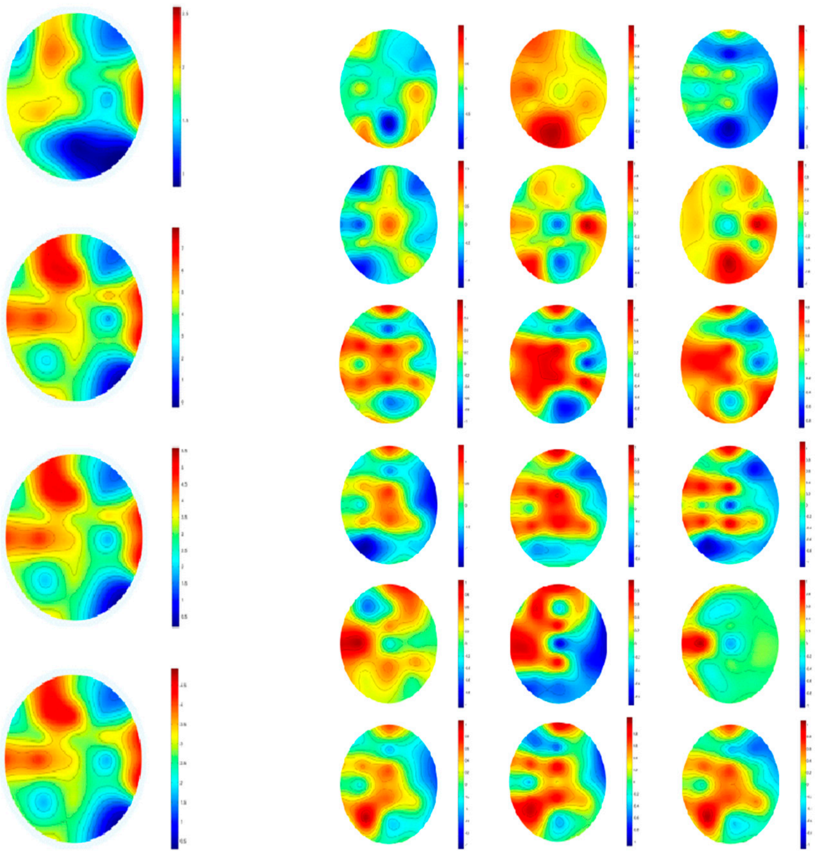

Supplementary Figure S4. Time-resolved topography of β-power during binaural beats stimulation. A. From the top to bottom figures shown exposures of brain activity after 5, 60, 120 and 250 sec of stimulation beginning, respectively. **B.** Topography of β-power in reading experiments for each AWS participant (baseline, 0 min and 10 minutes after).

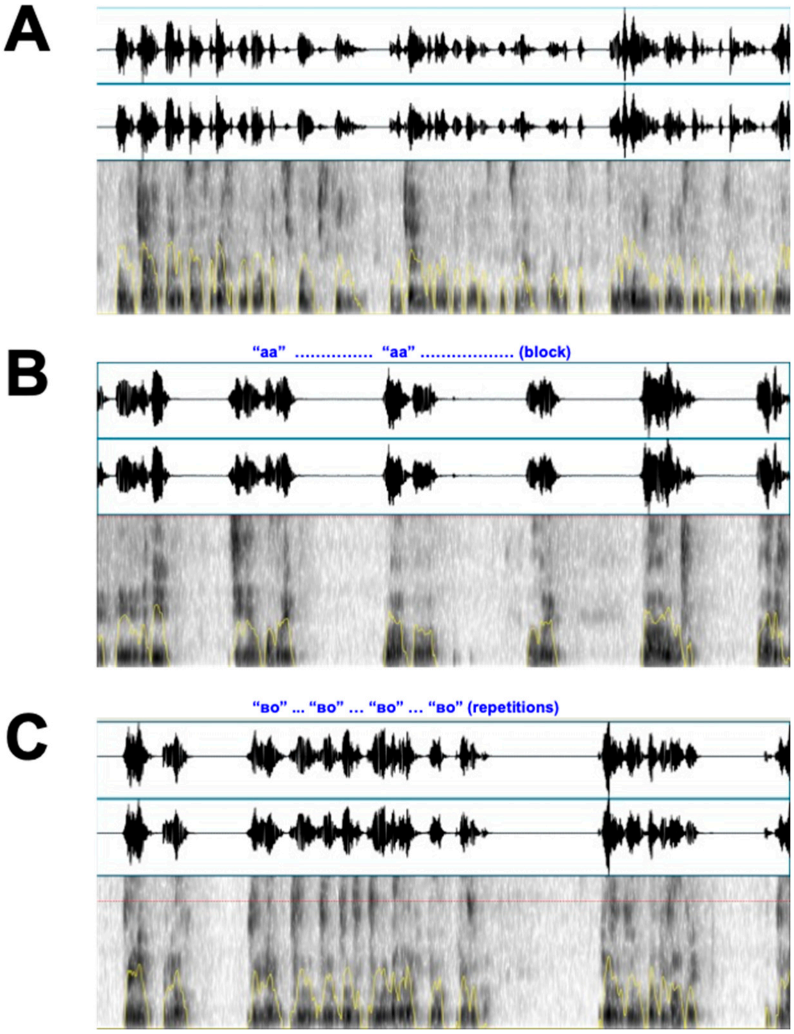

Supplementary Figure S5. Snippets of the raw speech signals. Raw signal and spectral characteristics of fluent speech (**A**), speech with blocking episode (**B**) and repetitions episode (**C**).

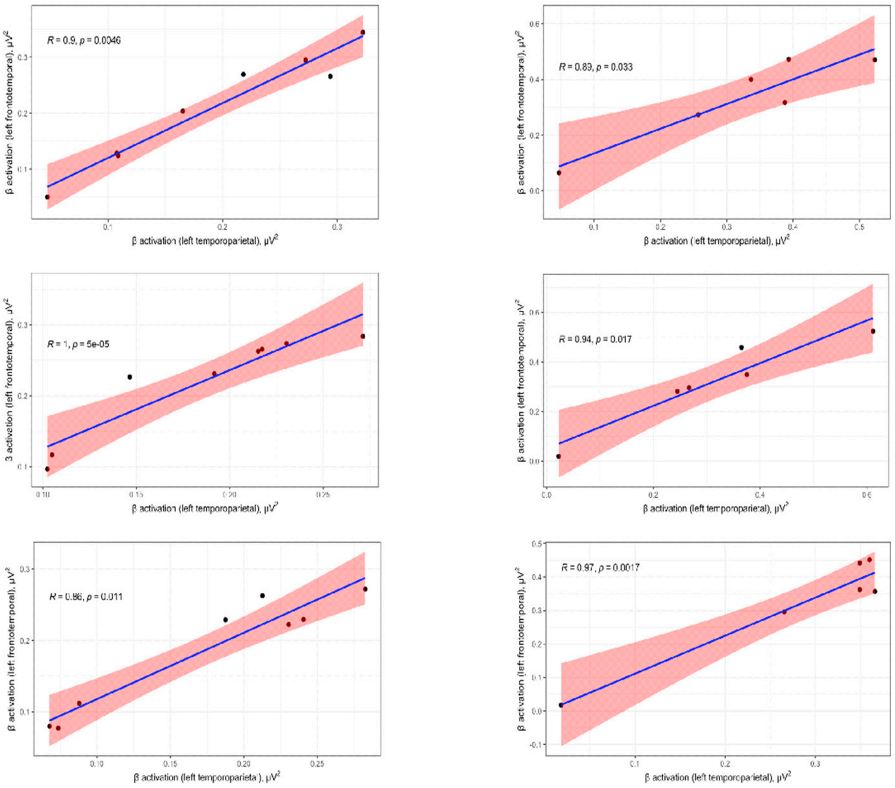

Supplementary Figure S6. Cross-sectional correlation between normalized activity the left temporoparietal (LTP) projection and left frontotemporal (LFT) area in AWS (A) and controls (B) before (top), immediately after the stimulation (middle), and 10 minutes later (bottom).

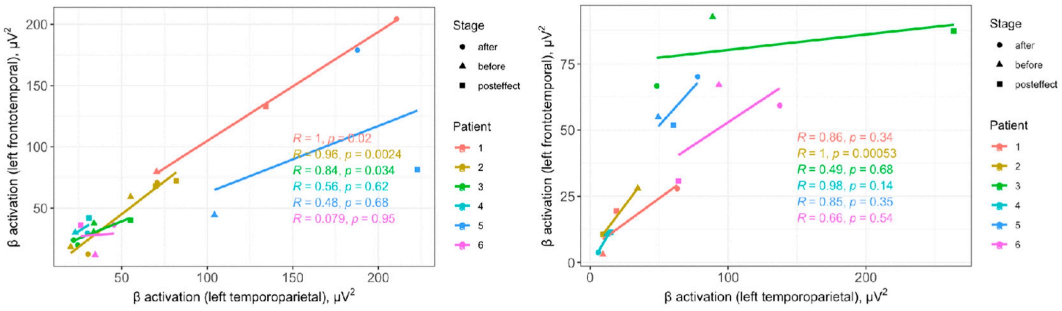

Supplementary Figure S7. Cross-sectional correlation between activity the left temporoparietal (LTP) junction and left frontotemporal (LFT) area in AWS (A) and controls (B) for each AWS participant.
